# Alkaline pH‐Driven Metabolic Plasticity of *Lactococcus lactis* FM03

**DOI:** 10.1111/1462-2920.70200

**Published:** 2025-11-06

**Authors:** Tamara A. L. Bendig, Tjakko Abee, Sjef Boeren, Eddy J. Smid, Oscar van Mastrigt

**Affiliations:** ^1^ Food Microbiology Wageningen University and Research Wageningen the Netherlands; ^2^ Wageningen University and Research Wageningen the Netherlands

**Keywords:** chemostat, high pH, lactic acid bacteria, metabolic shift, resource allocation

## Abstract

The dairy starter *Lactococcus lactis* shifts its metabolism from mixed‐acid fermentation to homolactic fermentation under anaerobic conditions as growth rates increase. Although its metabolism at acidic and neutral pH values is well‐researched, knowledge about lactococcal physiology under alkaline conditions remains limited. Here, we investigated how *L. lactis* subsp. *lactis* biovar diacetylactis FM03 adapts its metabolism and morphology at alkaline pH using lactose‐limited chemostat cultures at pH 6, 7 and 8. At alkaline pH, *L. lactis* FM03 shifted from energetically more favourable mixed‐acid fermentation towards homolactic fermentation at lower growth rates compared to pH 6, resulting in a 20% lower biomass yield despite an unchanged maintenance coefficient and maximum biomass yield per ATP. Proteome analysis revealed a 1.5 to 13.5‐fold downregulation of enzymes in the mixed‐acid fermentation pathway at alkaline pH, thereby reducing its metabolic capacity. Morphologically, *L. lactis* became more spherical at alkaline pH, reducing the surface‐to‐volume ratio and did not enlarge upon higher dilution rates. This morphological shift potentially limits substrate uptake, contributing to the lower maximum growth rate at pH 8. Our findings reveal new insights into pH‐driven metabolic plasticity and resource allocation in *L. lactis* and highlight opportunities for optimising fermentation processes under varying pH conditions.

## Graphical abstract

Alkaline pH induces a metabolic shift in *Lactococcus lactis* FM03 from mixed‐acid to homolactic fermentation, driven by proteome‐level constraints in the mixed‐acid branch. This shift reduces biomass yield despite unchanged bioenergetics. Moreover, alkaline pH changes cell morphology and prevents elongation at higher growth rates. These adaptations offer insights into pH‐dependent resource allocation and opportunities for optimising industrial fermentations.

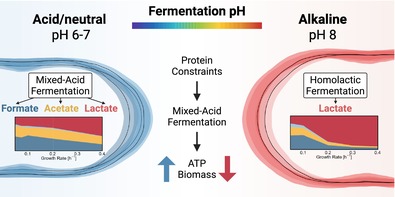

## 1 Introduction

Lactic acid bacteria (LAB) are important in starter cultures for various fermentation processes, spanning vegetable products like sauerkraut and kimchi to dairy products such as sour cream, yoghurt, buttermilk and cheese. Among these LAB, *Lactococcus lactis* stands out for its robustness and its contribution to product outcomes, due to its strong production of organic acids, mainly lactic acid. These organic acids are important for organoleptic and textural product properties as well as for food preservation by inhibition of undesired microorganisms.

The production of organic acids occurs through the reduction of pyruvate, which is formed as *L. lactis* derives its energy from glycolysis coupled with fermentation under anaerobic conditions. However, these fermentation products can vary as *L. lactis* can shift between homolactic or mixed‐acid fermentation, which is influenced by factors such as carbon limitation, oxygen availability and specific growth rate. At low specific growth rates or carbon‐starved conditions, *L. lactis* typically diverts the metabolic flux towards mixed‐acid fermentation (Thomas et al. 1979), resulting in the formation of formate, acetate and ethanol as metabolic end‐products, rather than primarily lactate. As the mixed‐acid branch generates more ATP per molecule of glucose consumed, this pathway is energetically more favourable compared to the homolactic branch. The control over the shift has been attributed to various reasons, such as metabolite levels, the NADH/NAD^+^ ratio (Garrigues et al. 1997), enzyme abundances (Melchiorsen et al. 2002) or the limited space in the cytoplasmic membrane available for solute transport systems (Zhuang et al. 2011), though the exact mechanism is still under debate (reviewed in Goel et al. 2012).

As a consequence of using either of these metabolic pathways (i.e., homolactic versus mixed‐acid), *L. lactis* acidifies its environment to a pH of 4. pH values below or above its optimal pH range of 6.0–6.9 (Hugenholtz et al. 1987; Parente et al. 1994) can heavily impact microbial physiology and the glycolytic rate, energy production and viability, despite the presence of molecular mechanisms to cope with pH changes. One factor that reduces biomass formation under acidic conditions is the higher inhibitory effect of weak organic acids such as lactic acid or acetic acid due to their higher accumulation in the cytoplasm and consequently uncoupling effect (Brul and Coote 1999; Mercade et al. 2000). In acidic environments, these organic acids are undissociated and diffuse passively across the cell membrane. So far, a dedicated lactic acid transporter has not been identified for *L. lactis;* thus, the intracellular accumulation might be caused mainly by passive diffusion. Inside the cell, where the pH is near neutral and therefore more alkaline, the acid dissociates, becomes trapped as an anion, accumulates and ultimately causes end‐product inhibition of growth. Further, the intracellular dissociation contributes to lowering the intracellular pH, causing proton stress and growth inhibition despite the presence of a residual carbon source (Loubiere et al. 1997; Mercade et al. 2000; Carpenter and Broadbent 2009).

Conversely, a higher extracellular pH potentially reduces lactic acid influx and intracellular accumulation and thus might lower end‐product inhibition. However, the growth behaviour at higher pH values with potentially lower organic acid inhibition remains to be elucidated. Despite extensive studies of the physiology of LAB, particularly *L. lactis* under neutral or acidic conditions, its metabolic behaviour under alkaline conditions remains largely unexplored. Although *L. lactis* is unlikely to be exposed to alkaline pH in natural habitats, it demonstrates surprising growth capabilities even at a pH value of 9.2 (Balows et al. 1992), some even up to 9.6 (Nomura et al. 2006), suggesting undiscovered strategies to cope with alkaline conditions. However, studies focusing on metabolic strategies in non‐alkaliphilic LAB to face alkaline conditions are rare, apart from a few proteomic studies (Rhee and Pack 1980; Sawatari and Yokota 2007; Hansen et al. 2016; Wu et al. 2020). Such studies could yield valuable insights into unknown metabolic coping strategies and metabolic plasticity with implications for both biotechnology and food fermentation, including methods to steer the metabolism of bacteria during fermentations to produce, for instance, altered flavour profiles or manage post‐acidification. For example, in *Lactiplantibacillus plantarum* and *Lactobacillus delbrueckii* subsp. *bulgaricus*, an increase in pH has been shown to shift their metabolism towards mixed‐acid fermentation (Rhee and Pack 1980; Tseng and Montville 1992). That is, for *L. plantarum* (Tseng and Montville 1992), a shift from pH 5.5 to 7.5 in a chemostat culture caused a fourfold increase in the specific production rate of acetate, while the specific production rate of lactate increased slightly.

In this study, we investigated the metabolic dynamics of *L. lactis* FM03 (van Mastrigt et al. 2017) under alkaline conditions in a pH‐controlled chemostat culture. We explored how pH and dilution rate impact growth parameters, the balance of mixed‐acid versus homolactic fermentation pathways and cell morphology. To gain insights into the mechanism underlying these effects, we integrated proteomic analyses to quantify the abundance of key enzymes involved in glycolysis as well as in mixed‐acid and homolactic fermentation. This approach allowed us to shed light on pH‐induced and growth rate‐dependent metabolic changes at the protein and metabolite levels and understand the metabolic shift caused by an alkaline environment.

## 2 Materials and Methods

### 2.1 Strain and Culture Conditions

This study was conducted with *L. lactis* subsp. *lactis* biovar diacetylactis FM03 (van Mastrigt et al. 2017). Prior to all chemostat cultivations, a −80°C stock was streaked on M17 (Difco, #218561) agar plates containing 1.5% bacteriological agar (Oxoid, #LP001113) and 0.5% (w/v) lactose (LM17). After an incubation period of 2–3 days at 30°C, one colony was used to inoculate 10 mL LM17 to generate a pre‐culture. This pre‐culture was incubated overnight at 30°C. All experiments were performed in a chemically defined medium (van Mastrigt et al. 2018) with the modifications of 0.24% (w/w) tri‐ammonium citrate, 1% tryptone (w/w) and 0.5% (w/w) lactose as main carbon and nitrogen sources.

### 2.2 Chemostat Cultivations

*L. lactis* was grown in bioreactors with a working volume of 0.5 L (Multifors, Infors HT) at a dilution rate between 0.05 and 0.4 h^−1^. The temperature was maintained at 30°C, the agitation speed was set at 200 rpm and anaerobic conditions were set by continuously flushing the headspace with nitrogen at a flow rate of 0.1 L/min. If signs of wall growth were observed, the agitation speed was increased to 300 rpm, which reliably prevented biofilm development. The pH was controlled and automatically adjusted by the addition of 5 M NaOH. The optical density at 600 nm was continuously monitored using an internal probe (EXcell 231, Exner Process Equipment). The bioreactor was inoculated at 1% (v/v) with a pre‐culture in LM17. After the culture reached the stationary phase, observed by a stabilisation of the optical density and base addition rate, the feed pump was started to continuously add fresh medium to the culture at a fixed rate corresponding to the desired dilution rates. The feed rate was continuously monitored based on the decline in the weight of the medium vessel and automatically adjusted by a soft sensor implemented in eve bioprocess platform software. The working volume was maintained by continuously removing culture broth at a fixed height corresponding to a 0.5 L working volume. Samples were taken after at least five volume changes in the chemostat mode and after maintaining a stable optical density as well as base addition rate. Steady states were established consecutively within the same continuous culture by altering either pH or dilution rate in a randomised order. For the chemostats with varying pH, the culture was returned to the initial pH value to verify reproducibility. The final samples were not included in data analysis. The experiments were planned for at least three independent biological replicates per pH. However, recurring prophage activation, especially during continuous cultures at pH 6, did not allow for the completion of the intended number of replicates. In such instances, data from at least two successful samples were included in the analysis.

### 2.3 Cell Viability Assessment

The optical density was determined with a spectrophotometer in appropriate dilutions. Viable plate counts were measured by diluting cell suspensions in peptone physiological salt (PPS, #P100.25.0009, Tritium Microbiologie) and spot‐plating appropriate dilutions on LM17 plates (Sieuwerts et al. 2008). The colonies were counted after 2 days of incubation at 30°C and the sum of 15 spots with 5 μL each was used to calculate the colony forming units (CFUs) mL^−1^. To measure the dry weight, 5–15 mL cell suspension from the bioreactor was passed through a pre‐weighed Supor PES membrane (#60301, Pall Corporation) with a pore size of 0.2 μm using a vacuum filtration unit. The samples were washed twice with approximately 20 mL Milli‐Q Millipore water and dried at 80°C for 3 days before weighing again on an analytical balance.

### 2.4 Analysis of Extracellular Metabolites

The extracellular metabolites acetate, acetoin, citrate, ethanol, formate, lactate and lactose were quantified by high‐performance liquid chromatography (HPLC). The samples were prepared by collecting the cell suspension from the bioreactor, followed by centrifugation at 4°C and 17,000*g* for 2 min. The resulting supernatant was stored at –20°C until analysis. For HPLC analysis, 5 mM sulphuric acid was used as the mobile phase at a flow rate of 0.6 mL/min. The separation was performed on an UltiMate3000 (Thermo Fisher Scientific) equipped with an Aminex HPX‐87H column (300 × 7.8 mm, Bio‐Rad) as a pre‐column at a column temperature of 40°C. Detection and quantification of metabolites were achieved with a refractive index detector (RefractoMax 520, ERC).

### 2.5 Rates and Maintenance Requirements

The ATP production rate (*R*_ATP_) was calculated based on the metabolite production rates using Equation (1), with the production rate *R*_*i*_ (mmol h^−1^) given by Equation (2). In Equation (2), *c*_*i*0_ and *c*_*i*_ refer to the concentration of component *i* in the medium and cell suspension, respectively, given in mM, while *v*_0_ refers to the flow rate in L/h.

(1) $$R_{\mathrm{ATP}}=R_{\text{lactate}}+R_{\text{ethanol}}+R_{\text{pyruvate}}+2\cdot R_{\text{acetoin}}+2\cdot R_{\text{acetate}}$$

With

(2) $$R_{i}=c_{i0}\cdot v_{0}-c_{i}\cdot v_{0}$$

The specific production rates were calculated by dividing the production rate *R*_*i*_ by the biomass (gDW).

A linear relationship was assumed between the dilution rate *D* and the steady state biomass‐specific ATP production rate (*q*_ATP_, in mmol gDW^−1^ h^−1^). A linear regression line was fitted to the data using the emmeans package in R. According to Equation (3), the maximum biomass yield on ATP (*Y*_X/ATP_^max^ in mol_gDW_ mol_ATP_^−1^) is the inverse of the slope of the regression line and the maintenance coefficient (*m*_ATP_ in mmol ATP·gDW^−1^ h^−1^) is the intercept.

(3) $$q_{\mathrm{ATP}}=\frac{D}{Y_{\begin{array}{c} \;X/{\mathrm{ATP}}^{\max}\\ \; \end{array}}\;}+m_{\mathrm{ATP}}$$

### 2.6 Proteome Sample Preparation

The proteomic samples were prepared and measured in biological quadruplicates per pH condition, obtained from independent chemostat runs at a fixed dilution rate of 0.2 h^−1^. The cell suspension (2 mL) was collected from the bioreactor and centrifuged at 4°C and 17,000*g* for 2 min. The pellet was flash‐frozen in liquid N_2_ and stored at −80°C until analysis. Before analysis, the pellet was washed twice in ice‐cold 100 mM Tris buffer pH 8 with Pierce Protease Inhibitor (#A32965, Thermo Scientific) and transferred to a Protein LoBind tube (Eppendorf). After the last washing step, the pellet was resuspended in 50 μL 100 mM Tris buffer pH 8 with Pierce Protease Inhibitor (#A32965, Thermo Scientific) and the cells were lysed on ice with three rounds of sonication with a soniprep 150 sonication probe (MSE) for 15 s on and 30 s off. The protein concentration was determined using Pierce BCA Protein Assay (#23225, Thermo Scientific) according to the manufacturer's instructions.

### 2.7 Proteome Sample Preparation and Analysis

The proteomic samples were further prepared using the protein aggregation capture (PAC) method (Batth et al. 2019). In short, the sample was reduced with 15 mM DTT while being incubated for 30 min at 45°C. Then, the proteins were unfolded with 6 M urea in 100 mM TRIS and alkylated in 20 mM acrylamide for 30 min at room temperature under constant medium shaking. The proteins were captured with two types of speed beads (#45152105050250 and #65152105050250, GE Healthcare) in a 1:1 ratio in acetonitrile. The proteins were bound to the speed beads by a 20 min incubation at room temperature, followed by two washing steps, first in 70% ethanol and then 100% acetonitrile. The proteins were digested overnight at room temperature under constant shaking with 100 μL sequencing‐grade trypsin (5 ng/μL) in 50 mM ammonium bicarbonate. To stop digestion and to lower the pH, 10% trifluoracetic acid was added and the sample was filtered over an Empore C8 filter into a 0.5 mL low‐binding Eppendorf tube. The remaining beads were washed with 50% acetonitrile and 50% formic acid in water and the washing fluid was filtered and combined with the digested protein sample in the low‐binding Eppendorf tube. The samples were concentrated and reconstituted into 50 μL using formic acid in water. Finally, the samples were measured by injecting 5 μL into a Vanquish Neo nanoLC—Orbitrap Exploris 480 MS/MS (Thermo Scientific) as described (Zheng et al. 2024). The MS data were analysed using the MaxQuant quantitative proteomics software package with the proteome of *L. lactis* FM03 (Genome assembly ASM214821v1) as the database and settings as described in (Liu et al. 2021).

### 2.8 Proteome Analysis

The analysis of the proteomes under different pH was conducted using normalised label‐free quantitation (LFQ) intensities from the MaxQuant result. A detected protein was included in the analysis if it appeared in at least three out of four biological replicates in at least one pH condition. Missing values were imputed by random sampling from a narrowed (0.3 *σ*) and downshifted (1.8 *σ*) normal distribution (Lazar et al. 2016). Proteins with at least a 1.5‐fold change (|log_2_‐fold change| ≥ 0.585) and a Tukey‐adjusted *p*‐value ≤ 0.05 (one‐way ANOVA with Tukey's *post hoc* test for multiple comparisons) were considered significantly different.

### 2.9 Microscopic Approach to Determine Cell Sizes

To obtain the single‐cell dimensions, we visualised cells in a fresh cell suspension fixed using a thin 2% (w/v) agar pad on a microscope slide with a coverslip under phase contrast settings using a BX 41 microscope (Olympus). 1000‐fold magnification was reached with a UIS‐2 PLANC ×100 PH3 oil immersion lens (Olympus) and pictures were taken with an XC 30 digital camera (Olympus) with a ×0.63 magnifier tube and the software Olympus CellB version 3.5. The images were pre‐processed in ImageJ version 1.53 t (Rasband 2011) by first converting them to 8‐bit greyscale, setting a scale where the known pixel size equals 0.0345 μm and correcting for uneven illumination and removing background noise with the rolling ball background subtraction with a rolling ball radius of 50 pixels. Then, the images were imported into MicrobeJ version 5.13 p(9) (Ducret et al. 2016) and the cell dimensions were acquired by detecting cells with an area between 0.7 and 3.5 μm^2^ (Sieuwerts et al. 2008), a length over 0.8 μm, a width between 0.5 and 2 μm and with a higher circularity than 0.65. Further, we excluded cells touching the edges, did not allow for branching and re‐segmented rejected particles.

Per cell *i*, the cellular volume (*V*_*i*_ in μm^3^) was calculated with Equation (4) and the cell surface area (SA_*i*_ in μm^2^) with Equation (5), assuming that the cells are cylindrical with two half‐spheres as caps. Since cell length (*l*_*i*_) was defined as the length along the medial axis of the cell, in some cases (< 5%), the measured length of one cell within a doublet was smaller than its width (*w*_*i*_). Cell measurements where the length was greater than the width were excluded from analysis.

(4) $$V_{i}=\pi \cdot \frac{w_{i}^{2}}{4}\cdot \left(l_{i}-\frac{w_{i}}{3}\right)$$

(5) $${\mathrm{SA}}_{i}=\pi \cdot w_{i}\cdot \left(l_{i}-w_{i}\right)+\pi {\cdot w}_{i}^{2}$$

The fraction of singlets and doublets was calculated using data from MicrobeJ, based on the occurrence of the same parent cell. The accuracy of the segmentation of parent cells was verified manually. Cell chains consisting of three cells or more were counted manually and added to the data.

### 2.10 Statistical Tests

The pairwise comparisons of means across the three pH levels were performed using a pairwise *t* test as implemented in RStudio Build 369. The Bonferroni correction was applied to correct *p*‐values for multiple comparisons.

## 3 Results

### 3.1 Biomass Accumulation

**FIGURE 1:**
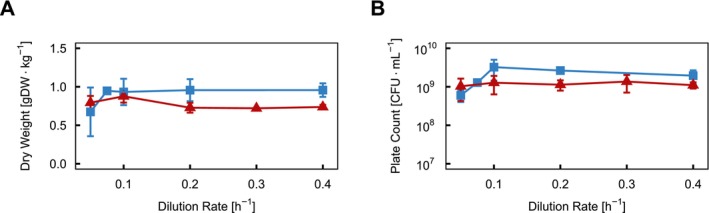
Steady‐state biomass accumulation of *Lactococcus lactis* FM03 in a lactose‐limited chemostat cultivation at pH 6 and 8 as a function of dilution rate. (A) The biomass is expressed as cell dry weight (gDW kg^−1^) and (B) as viable plate counts (CFU mL^−1^) in a chemostat at dilution rates ranging from 0.05 to 0.4 h^−1^. The pH was set to 6 (blue squares) or 8 (red triangles). Each data point shows the average with standard deviation of at least three independent biological replicates, except for the data points at pH 6 with a dilution rate of 0.05, 0.075 and 0.1 h^−1^, for which only duplicate measurements were available.

*L. lactis* subsp. *lactis* biovar diacetylactis FM03 was grown anaerobically in lactose‐limited chemostats at dilution rates between 0.05 and 0.4 h^−1^ on a chemically defined medium containing lactose as well as citrate. During cultivation, the pH was kept constant at pH 6 or 8. A dilution rate of 0.4 h^−1^ was close to the maximally feasible dilution rate at pH 8 before cell washout occurred, but still allowed for a steady state as indicated by the high biomass concentration. For reference, the maximal feasible dilution rate for pH 6 is reported to be around 0.7 h^−1^ (Goel et al. 2015). At both pH values, the cell dry weight (Figure 1A) and plate count (Figure 1B) stayed almost constant across the range of dilution rates. The differences between pH 6 and 8 were more pronounced at dilution rates higher than 0.1 h^−1^. At pH 6, *L. lactis* FM03 reached on average 0.94 ± 0.09 gDW/kg, neglecting the lowest dilution rate and slightly less at pH 8 with 0.77 ± 0.08 gDW/kg. The average plate counts were 1.8‐fold lower at pH 8.

### 3.2 Metabolite Distribution in Dependence on pH

**FIGURE 2:**
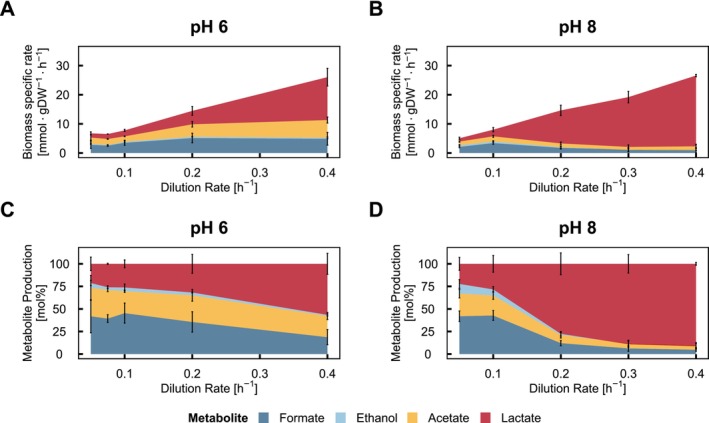
Metabolite flux distribution by *Lactococcus lactis* FM03 in a lactose‐limited chemostat cultivation at pH 6 and pH 8. The biomass specific production rates in mmol·gDW^−1^ h^−1^ were measured in a chemostat at dilution rates ranging from 0.05 to 0.4 h^−1^ at pH 6 and 8. (A, B) Biomass‐specific metabolite production rates at pH 6 and 8, respectively. (C, D) Normalised metabolite production at pH 6 and 8, respectively. Formate: dark blue, ethanol: bright blue, acetate: yellow, lactate: red. Error bars represent the standard deviation of at least three replicates for pH 8 and at least two replicates for pH 6.

To explore the growth rate‐dependent metabolic shift under alkaline conditions, the extracellular metabolite concentrations were measured to estimate flux distribution over the homolactic and mixed‐acid branches (Figure 2). At low dilution rates (*D* ≤ 0.1 h^−1^), around three‐quarters of the metabolic end products originated from the mixed‐acid branch for both pH conditions. While the specific production rates of mixed‐acid branch products at pH 6 increased between dilution rates of 0.1 and 0.2 h^‐1^ and stayed constant at a dilution rate of 0.2 and 0.4 h^−1^, they declined slightly for pH 8 conditions. The specific production rate of lactate increased progressively with increasing dilution rates for both pH conditions. At the highest tested dilution rate (0.4 h^−1^), lactic acid accounted for around 90% of all products at pH 8, compared to only 50% at pH 6. The restricted flux through the mixed‐acid branch caused a significantly earlier and more pronounced metabolic shift to homolactic fermentation at pH 8, even if a lower feasible maximal growth rate is considered.

### 3.3 pH‐Dependent Bioenergetics

The maintenance coefficient *m*_ATP_ and the maximum biomass yield on ATP (*Y*_*X*/ATP_^max^) were calculated from the linear dependence of the growth rate and the biomass‐specific ATP production rate at dilution rates between 0.05 and 0.4 h^−1^ at pH 6 and 8 (Figure S1A). No significant differences were found between the maintenance coefficients and the maximum biomass yield on ATP calculated from pH 6 and 8 (Table 1).

**TABLE 1:** Maintenance coefficient and maximum biomass yield on ATP.

pH	*m*_ATP_ (mmol_ATP_·gDW^−1^ h^−1^)	*Y*_x/ATP_^max^ (gDW mol_ATP_^−1^)
6	1.22 ± 1.03	15.24 ± 1.04
8	0.65 ± 0.90	15.67 ± 0.94

### 3.4 Effect of pH on Culture Density, Viability and Chain Formation

**FIGURE 3:**
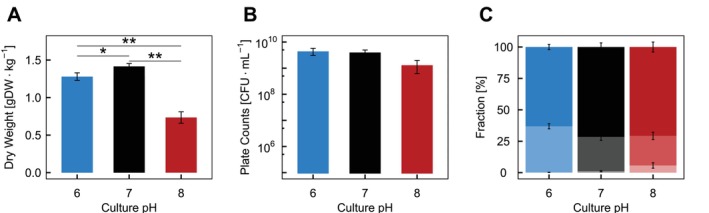
Effect of pH on culture density and chain length in a chemostat culture with a dilution rate of 0.2 h^−1^ grown at pH 6 (blue), pH 7 (black) and pH 8 (red). (A) Biomass concentration in dry weight in gDW kg^−1^. (B) Culturable cell counts determined by spot plating on LM17 agar plates in CFU mL^−1^. (C) Chain length fractions. Darkest shade: singlets, medium shade: doublets, lightest shade: chains with three cells or more. The chain fractions were determined from 2575 cell chains at pH 6, 3931 cell chains at pH 7 and 1751 cell chains at pH 8. Cells chains containing more than two cells were counted manually, while the fraction of chains with up to two cells were calculated by MicrobeJ. The asterisks represents the adjusted statistical significance levels with *p ≤ 0.05 and **p ≤ 0.01, calculated using a paired *t* test with Bonferroni correction. The data was calculated from four independent experiments to calculate the dry weight and the culturable cell counts and three independent experiments for the chain length.

To get further insights into its physiology at alkaline pH, *L. lactis* FM03 was grown at pH 6, 7 and 8 in anaerobic lactose‐limited chemostat cultures with a fixed dilution rate of 0.2 h^−1^. The growth performance was assessed by analysing the cell dry weight and CFUs. The cell dry weight declined significantly from pH 6 and 7 to pH 8 (1.28 ± 0.05 and 1.42 ± 0.04 to 0.74 ± 0.08 gDW kg^−1^; Figure 3A), with a corresponding decrease in OD_600_ from 3.66 ± 0.1 and 3.86 ± 0.08 to 2.24 ± 0.28 (Figure S2A). Their similar declining trend suggested that alkaline pH reduced the overall culture density and did not strongly skew the relation between OD_600_ and dry weight measurements (Figure S2B). Similarly, the viable plate count measured in CFU mL^−1^ declined at pH 8 (Figure 3B). Interestingly, pH 6 and 7 supported more than three times the amount of CFU mL^−1^ (Figure 3B) and approximately twice for CFU mL^−1^·OD_600_^−1^ (Figure S2C) compared to pH 8. Microscopic analysis revealed that for pH 6, 7 and 8, the majority of cells appeared as singlets and around 30% appeared as doublets (Figure 3C). Additionally, chaining slightly increased for *L. lactis* FM03 at pH 8, with cell chains containing more than two cells constituting 0.2% ± 0.1% at pH 6, 1.1% ± 0.5% at pH 7 and 5.7% ± 2.2% at pH 8. The manual counts for cell chains with more than two cells are given in Table S1. This small change in cell chaining was too low to account for the reduction of CFU at pH 8 and, therefore, suggested that the reduced cell density stemmed from other factors.

### 3.5 pH‐Dependent Morphology

**FIGURE 4:**
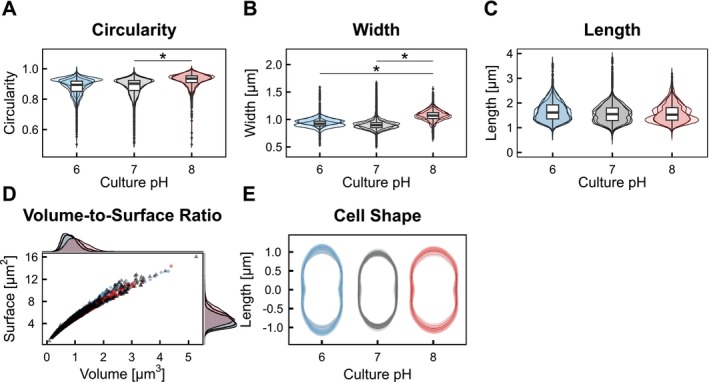
Effect of pH on the cell size of *Lactococcus lactis* FM03 in a chemostat with a fixed dilution rate of 0.2 h^−1^. The cell sizes were analysed from microscopic images using the ImageJ plugin MicrobeJ (version 13p). Shown are the results of independent chemostat runs, presented in different shades of the respective colour. The asterisks represents the adjusted statistical significance level with *p ≤ 0.05, calculated using a paired *t* test with Bonferroni correction. (A) Circularity, (B) cell width calculated by the mean of values measured along the medial axis of a cell, (C) length of the medial axis, (D) volume to surface ratio. pH 6 blue circles, pH 7 black triangles and pH 8 red squares, (E) overlay of bacterial cell outlines with standard deviation.

Changes in the cell size can affect metabolic rates, for example, nutrient uptake. Moreover, differences in optical density could be linked to changes in cell size. Therefore, we measured the cell sizes of *L. lactis* FM03 at different pH conditions. *L. lactis* typically displayed an ovoid cell shape with a slight elongation. Elevation of the pH caused a shift in their morphology towards visibly rounder cells. To quantify the morphological changes, we analysed single‐cell dimensions on microscopic images with a 1000‐fold magnification. At alkaline pH, the cells' circularity increased due to a significant widening of the cells, from 0.93 ± 0.08 μm at pH 6 and 0.90 ± 0.1 μm at pH 7 to 1.07 ± 0.10 μm at pH 8 (Figure 4B). The cell length did not change significantly and remained constant at around 1.6 μm (Figure 4C). These changes resulted in a 20% and 34% increase in cell volume at pH 8 compared to pH 6 and 7, while the surface area increased only 10% and 19%, respectively. Consequently, the surface‐volume ratio decreased at pH 8 to about 90% of its value at pH 6. Moreover, cells cultivated at pH 8 showed a broader distribution of cell volumes compared to pH 6 and 7, with more data points at a slightly higher volume. Such differences in morphologies can influence OD_600_ measurements. While the ratio between dry weight and optical density remained constant (Figure S2B), the viable plate count per OD_600_ unit tended to be lower at pH 8 (Figure S2C), indicating fewer culturable cells per OD. However, this trend was statistically not significant and should be interpreted with caution. Interestingly, while increasing the dilution rate caused the cells to become longer and wider at pH 6, neither the cell length nor the cell width were affected by the growth rate at pH 8 (Figures S3 and S4).

### 3.6 pH‐Dependent Alterations in Central Metabolism

**FIGURE 5:**
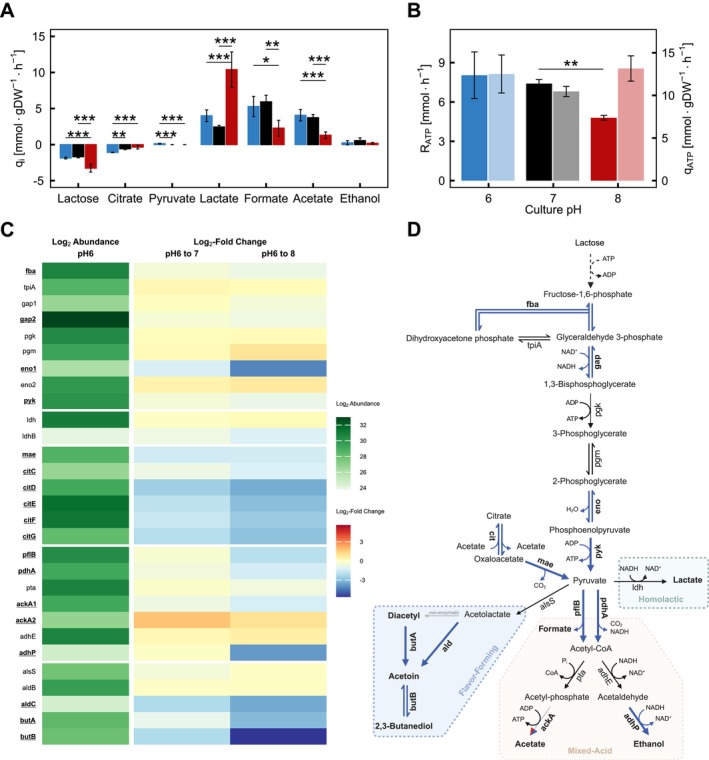
Effect of pH on the specific metabolite production rates, ATP production and protein expression. The metabolites were measured in cell‐free supernatant from the chemostat with a fixed dilution rate of 0.2 h^−1^. For (A, B): pH 6 shown in blue, pH 7 in black and pH 8 in red. (A) Specific production rates of metabolites. (B) ATP production rate (*R*_ATP_) (darker shade) and biomass specific ATP production rate (*q*_ATP_) (brighter shade) calculated from the metabolite production rates. (C) Protein abundance. Proteins with significantly altered expression are written underlined and in bold. The protein abundance (first column) and fold‐change between pH 6 and 7 (second column) and 6 and 8 (third column) are LOG_2_‐transformed. (D) Schematic display of the glycolytic pathway with citrate metabolism, flavour‐forming branch, mixed‐acid branch and homolactic branch. Proteins with significantly altered are written in bold and either blue if they are downregulated or red if they are up‐regulated at pH 8 compared to pH 6. A protein is considered significantly altered with an absolute fold change of at least 1.5, and ANOVA as well as an adj. *p*‐Value ≤ 0.05 between pH 6 and 8. Stoichiometries of metabolites are neglected in this overview. These data are provided in Table S2. The asterisks represents the adjusted statistical significance levels with *p ≤ 0.05, **p ≤ 0.01 and *** = *p* ≤ 0.001, calculated using a paired *t*‐test with Bonferroni correction. cit is the abbreviation for the complex consisting of citC, citD, citE, citF and citG and ald includes aldB (stable) and aldC (downregulated).

To dive deeper into the physiological impact of alkaline pH on *L. lactis* FM03, we studied the metabolism at a fixed dilution rate of 0.2 h^−1^ at pH 6, 7 and 8. At a dilution rate of 0.2 h^−1^ and pH 6, around three‐quarters of the fermentation products originated from the mixed‐acid branch and the specific production rates for lactate, formate and acetate were similar in the chemostat culture at pH 6 and 7 (Figure 5A). In contrast, the contribution of the mixed‐acid branch dropped to ~25%, and consequently, lactate dominated with ~75% of the products at pH 8 (Figures 2B and 2D). Thus, alkaline pH impaired the use of the mixed‐acid branch, effectively rerouting pyruvate into lactate, reflected by an approximately doubled specific lactate production rate and a significantly declined (~75%) specific production rates of formate and acetate, compared to pH 6 and 7 (Figure 5A). In contrast, the specific production rate of ethanol remained similar at each pH value. The shift in the metabolic route comes with an energetic burden: the mixed‐acid branch after the Embden‐Meyerhof‐Parnas pathway yields 6 mol ATP mol^−1^ lactose, whereas the homolactic branch produces 4 mol. Consequently, the ATP production rate is lower at pH 8 compared to 6 and 7 (Figure 5B). However, the lower biomass at pH 8 and the increased flux shown by a significantly increased specific lactose consumption and lactate production rate compensate for the lower ATP yield. This resulted in a similar biomass‐specific ATP production rate at all tested pH levels (Figure 5B).

### 3.7 Proteomic Adjustments in Primary Metabolism to Alkaline pH

In order to assess if changed protein levels could account for the metabolic shift, we analysed the proteome for cells growing at a dilution rate of 0.2 h^−1^ at pH 6, 7 and 8. We found significant changes in protein abundances in pathways of the central metabolism, as well as the mixed‐acid, homolactic branch and flavour‐forming branch. However, the extent of the influence of pH on protein abundance differed between branches. Despite the higher specific lactose consumption rate at pH 8 (Figure 5A), the abundance of glycolytic enzymes remained stable or declined modestly (≤ 1.7‐fold) for three enzymes and strongly (18‐fold) for Eno1 compared to pH 6. Only Eno1 was downregulated at pH 8 compared to pH 7 (Figure 5C). In addition, for enzymes of the homolactic branch, the higher abundant l‐lactate dehydrogenase (Ldh) was not significantly differentially expressed, while the lower abundant isoform (LdhB) was more than two‐fold downregulated compared to pH 6, yet not significantly (*p* = 0.06). This indicated very limited pH‐induced changes in the protein abundances in glycolysis as well as the homolactic branch. In contrast, most enzymes of the mixed‐acid branch were strongly downregulated (up to 13.5‐fold) except for Pta, AdhE and AckA2. In addition, citrate metabolism (CitCDEFG) and the reactions involving diacetyl, acetoin and 2,3 butanediol in the flavour‐forming branch (AldC, ButA and ButB) were downregulated in a pH‐responsive manner, with a correlation coefficient *R* ^2^ above 0.93 showing a negative linear trend with increasing pH. The citrate transporter CitP was only detected in samples at pH 6 and fell below the detection limit for the other pH values. Thus, pH‐dependent expression was also expected for the citrate transporter, with reduced expression at higher pH values (van Mastrigt et al. 2018).

## 4 Discussion

Bacteria can adapt and thrive across diverse environments by adjusting and fine‐tuning their physiology. It is well known that the important dairy starter *L. lactis* can shift its metabolism between homolactic and the energetically more favourable mixed‐acid fermentation under anaerobic conditions. While the effects of substrate limitation and growth rate on fermentation pathways are typically studied under neutral to acidic pH, the impact of alkaline pH remains largely unexplored.

This study investigated how pH (pH 6, 7 and 8) and growth rates influence the physiology of *L. lactis* subsp. *lactis* biovar diacetylactis FM03 in lactose‐limited chemostat cultures. We focused on metabolic strategies, biomass yield, cell morphology and the proteomic underpinning of the observed changes in metabolism.

### 4.1 Cell Morphology

Lactococci can alter their cell shape in response to external factors such as nutrient availability and growth rate (Ercan et al. 2013; Taheri‐Araghi et al. 2015). Larger cells are typically associated with faster growth in *L. lactis* (Bachmann et al. 2013). This study confirms an increase in cell length and width for *L. lactis* FM03 at pH 6 upon higher dilution rates. In contrast, if the pH was elevated to pH 8, the cell sizes remained constant across all measured dilution rates (Figures S3 and S4). Focusing on the dilution rate of 0.2 h^−1^, we observed a change in cell shape upon a shift in pH to 8. While the cells are slightly rod‐shaped (ovoid) at acidic to neutral pH values, the cell width increased significantly under alkaline conditions, rendering them more spherical. This change in morphology and roundness lowers the surface area to volume ratio (S/V ratio) at alkaline pH. Although cells at pH 8 have a lower S/V ratio and do not enlarge with increasing growth rates, they still fully consumed lactose at all tested dilution rates. This indicates that the lactose uptake rate was not limiting growth up to a dilution rate of 0.4 h^−1^. However, the reduced S/V ratio combined with the lack of size expansion could limit lactose uptake at higher growth rates, thereby reducing the maximum growth rate at pH 8.

While alkaline pH also slightly increased cell chaining (≥ 3 cells per chain), these represented only a small fraction of the overall population. Therefore, this chaining does not explain the reduced CFU at pH 8 (Figure 3). Instead, this might be explained by the larger cell sizes at pH 8 or could be an indication of an increase in non‐viable or viable‐but‐not‐culturable cells.

### 4.2 Growth at Different pH

*L. lactis* FM03 achieved the highest biomass yield in terms of dry weight at pH 7, with only a minor decrease at pH 6, consistent with the optimal growth pH range of 6.0–6.9 as described in the literature (Hugenholtz et al. 1987; Parente et al. 1994). In contrast, elevating the pH by one unit to pH 8 reduced the biomass yield (Figure 1, Figure 3A) at the same growth rate, while the maintenance coefficient and maximum biomass yield per ATP were similar at pH 6 and 8 (Table 1). This indicated that the energy requirement for basic cellular functions remained unaffected, and they coped well with alkaline conditions. The lower biomass yield is the result of lower ATP production despite the same nutrient composition in the medium (Mira et al. 2022).

### 4.3 Metabolic Shifts Under Alkaline Conditions

The lower energy production could stem from the pronounced shift from mixed‐acid to homolactic fermentation under alkaline conditions. The shift from mixed‐acid to homolactic fermentation is more pronounced and occurs at much lower specific growth rates compared to pH 6 (Figures 2 and 5). A pH‐dependent metabolic shift towards homolactic metabolism was previously described in literature under acidic conditions, if the pH was lowered to pH 4.5 (Sánchez et al. 2008) or 5.5 (O'Sullivan and Condon 1999) in a chemostat, but an explanation for the origin of the shift was not given. Here, we showed that this shift could be the result of a restriction of the flux through the mixed‐acid branch, as the mixed‐acid branch fluxes do not increase with higher dilution rates. This suggests that the mixed‐acid pathway was operating at or near its maximal capacity, even at lower growth rates. Consequently, *L. lactis* FM03 routes the residual pyruvate into lactate formation—causing the shift to homolactic metabolism.

Goel et al. (2015) demonstrated that glycolytic enzymes and Ldh typically operate in overcapacity, with a weak connection between enzyme abundances, which are higher than necessary and the actual flux. In contrast, the PflB and Adh both operate close to their capacity and the corresponding fluxes can thus be restricted by enzyme abundances (Melchiorsen et al. 2002; Goel et al. 2015). At a dilution rate of 0.2 h^−1^, raising the pH from 6 to 8 mildly repressed enzymes of the lower glycolysis (below Fba) in a pH‐dependent manner, while enzymes of the upper glycolysis and the higher expressed Ldh isoform remain unchanged. Despite the restricted enzyme abundance, the specific flux through glycolysis was higher at pH 8 (Figure 5A), reflecting the overcapacity. In contrast, the proteins of the mixed‐acid branch sharply decreased in abundance at pH 8, causing a much lower capacity through those enzymes. In addition, the pyruvate formate‐lyase‐activating protein (PflA) decreased linearly with higher pH (1.43‐fold between pH 6 and 8), further limiting the activity of PflB and therefore capping acetate formation (Wagner et al. 1992). Goel et al. (2015) also showed lower enzyme abundances for PflB and Adh upon higher dilution rates. If this is also the case at alkaline pH, this could explain why our initially already restricted flux of the mixed‐acid branch could be even more restricted at dilution rates of 0.3 and 0.4 h^−1^ (Figure 2B), which is close to the maximally feasible dilution rate at pH 8 before washout occurred (data not shown). The restriction of capacity in the mixed‐acid branch forces the excess pyruvate into homolactic fermentation, also referred to as overflow metabolism. This rerouting has the energetic consequence of a significantly lower ATP production rate compared to mixed‐acid fermentation (Figure 5B), leading to a lower cell density at the same growth rate at pH 8 with the same amount of lactose (Figure 3).

Not only does the enzyme abundance but also the environmental pH define the functionality and the rate of enzymes. Nevertheless, the observed shift is unlikely to be driven by enzyme activity loss due to pH. For stationary‐phase lactococci, the pH gradient (ΔpH) diminishes at higher external pH (Molina‐Gutierrez et al. 2002) and approaches zero at pH 8 (Poolman et al. 1987; Breeuwer et al. 1996; Molina‐Gutierrez et al. 2002; Hansen et al. 2016). As the ΔpH is typically higher in growing cells compared to stationary cultures (O'Sullivan and Condon 1999), we can therefore assume that at an environmental pH of 8, the intracellular pH is close to 8. Generally, most glycolytic enzymes (e.g., phosphofructokinase Pfk and pyruvate kinase Pyk) and the Ldh remain active at pH 7–8 and even have optimal activity values within this range (Tuominen and Bernlohr 1971; Mou et al. 1972; Collins and Thomas 1974; Fordyce et al. 1982; Gaspar et al. 2007). In addition, two closely related LAB, *L. delbrueckii* and *Streptococcus thermophilus* , showed the highest utilisation of glucose and production of lactate at pH 8 in permeabilised cells (Arioli et al. 2010, 2017), indicating robustness of glycolysis and the homolactic branch towards pH 8. No data were found for pH‐dependent enzyme activities for *L. lactis* in the mixed‐acid branch. However, since we measured similar biomass‐specific production rates for mixed‐acid products at pH 6 and pH 8, at dilution rates of 0.1 h^−1^ and lower, we hypothesise that high pH does not inactivate PflB in *L. lactis* FM03 to a high extent, and the metabolic shift was mainly driven by changes in enzyme abundance.

While the activities of enzymes of the homolactic branch, as well as the mixed‐acid branch, seem pH‐stable, the flavour‐forming branch seems to be restricted by alkaline pH. Both the diacetyl reductase (ButA) and the acetoin reductase (ButB) show a pronounced decline in activity above pH 4.5 (Cachon and Diviés 1994). The pH‐restricted catalytic activity combined with their inherently low enzyme abundance points towards a severely restricted capacity, explaining why products of the flavour‐forming pathway were undetectable at pH 8.

The above considerations mainly address how *L. lactis* shifts between homolactic and mixed‐acid fermentation metabolism, but not why. By including protein synthesis costs into genome‐scale metabolic models, one model (Chen et al. 2021) showed that homolactic fermentation is more efficient at higher growth rates, while mixed‐acid fermentation is more efficient at lower growth rates. However, this resource allocation hypothesis is not in line with the overcapacity of the enzymes in glycolysis and the homolactic fermentation pathway. This indicates that *L. lactis* might not have optimised its resource allocation for growth under sugar‐limited conditions, as also suggested by a gradual shift to mixed‐acid fermentation upon prolonged cultivation in glucose‐limited chemostats at a dilution rate of 0.5 h^−1^ (Price et al. 2019). This could be explained by the lifestyle of LAB, which thrive in nutrient‐rich environments where they inhibit other microorganisms by fast acidification (Kleerebezem et al. 2020). In this view, overcapacity of glycolysis and the homolactic fermentation can be seen as preparedness without cost, as nutrients are abundant.

During rapid nutrient uptake, maintaining a balanced state, for example, in the upper and lower glycolysis, is crucial. For example, a study in yeast demonstrated that under high sugar influx, a subpopulation of cells experiences a mismatch between upper and lower glycolysis, leading to an imbalanced metabolic state with accumulation of intermediates, a constant low level of ATP and subsequently growth arrest (van Heerden et al. 2014). This imbalance can be avoided in yeasts by transiently diverting flux through trehalose metabolism, which acts as a futile cycle that stirs glycolysis into a balanced steady state. While *L. lactis* does not rely on trehalose cycling, a similar principle might apply for the NAD^+^ regeneration. The homolactic branch provides a rapid and equimolar route to regenerate NAD^+^, minimising the risk of a redox imbalance and/or ATP depletion as a result of a decreased flux through the lower glycolysis. In contrast, the mixed‐acid branch is less reliable, as only the ethanol branch regenerates NAD^+^, making it not stoichiometrically coupled to glycolysis and potentially problematic at high fluxes. Additionally, higher ATP production from the acetate branch may lead to ATP accumulation, which can inhibit glycolysis. Taken together, the homolactic branch is a simple yet fast and robust way to prevent metabolic imbalance.

### 4.4 Proton‐Consuming Reactions and Citrate Metabolism

At high intracellular pH, the amount of free protons becomes extremely scarce. Based on our cell volume measurements, we estimated around 7 free protons at an intracellular pH of 8. Despite the high buffering capacity in *L. lactis* by (organic) phosphates to absorb pH fluctuations (Levering et al. 2012; Poolman 2023), proton‐consuming reactions, such as decarboxylation reactions, should likely be minimised to prevent further alkalinisation of the intracellular pH. In addition, although the high intrinsic buffer capacity prevents local pH shifts, an abundance of proton‐consuming reactions might exhaust the buffer capacity and increase the energetic costs required for maintaining cytoplasmic pH homeostasis (Padan et al. 2005; Slonczewski et al. 2009). This is in line with the observed downregulation of citrate metabolism and acetoin production at alkaline pH. Both processes are important to alkalinise the cytosol under acidic stress conditions by consuming protons (Hugenholtz et al. 1993; Zuljan et al. 2014; Cesselin et al. 2018). We observed a strong negative correlation between pH and citrate consumption rates (Figure 5), as well as the abundance of related proteins (Mae, CitCDEFG in Figure 5), consistent with prior studies showing the induction of citrate metabolism and the citrate permease only under acidic conditions (Hugenholtz et al. 1993; García‐Quintáns et al. 1998; van Mastrigt et al. 2018). Moreover, the α‐acetolactate decarboxylase was downregulated more than tenfold at pH 8, indicating that proton consumption might indeed be disadvantageous under high pH conditions.

## 5 Conclusion

Despite the ability of *L. lactis* to grow even up to a pH value of 9.2, growth at pH 8 had an impact on physiology, metabolism and morphology. *L. lactis* FM03 shows a notable plasticity in its metabolism at pH 8 and reallocates a higher portion of its metabolic flux towards the less energy‐efficient homolactic branch. Proteome data indicated a downregulation of almost all proteins in the mixed‐acid pathway, which forces pyruvate through the homolactic branch and thus causes the metabolic shift. As the homolactic pathway yielded less ATP, less biomass was formed and consequently, the flux through glycolysis increased. As cell size did not increase at higher dilution rates at pH 8, membrane space may have become restricted, which could have limited lactose uptake rates and thus lowered the maximal growth rate. Overall, understanding how *L. lactis* fine‐tunes its metabolism under different pH conditions contributes to a broader understanding of the adaptability of bacteria and has potential implications for optimising industrial fermentation processes where pH control is crucial.

## Author Contributions

T.B.: writing – original draft, investigation, methodology, conceptualization, data curation, software, formal analysis, validation, visualization, writing – review and editing. T.A.: writing – review and editing, funding acquisition, supervision. S.B.: formal analysis, software, methodology. E.J.S.: supervision, project administration, funding acquisition, conceptualization, writing – review and editing, resources. O.M.: project administration, supervision, funding acquisition, conceptualization, writing – review, corresponding author

## Conflicts of Interest

The authors declare no conflicts of interest.

## Supporting information


**Data S1:** emi70200‐sup‐0001‐Supinfo.pdf.

## Data Availability

The data that support the findings of this study are openly available in the PRIDE partner repository at www.ebi.ac.uk/pride, reference number PXD065893.
