# Supplementary material for: Alkaline pH‐Driven Metabolic Plasticity of Lactococcus lactis FM03
Source: Environ Microbiol. 2025 Nov 6;27(11):e70200. doi: 10.1111/1462-2920.70200 (PMC12592913; doi:10.1111/1462-2920.70200)
Supplement: Supplementary file 1 — Data S1: emi70200‐sup‐0001‐Supinfo.pdf. [file EMI-27-e70200-s001.pdf]

Alkaline pH-driven metabolic plasticity of *Lactococcus lactis* FM03

Tamara A. L. Bendig[a], Tjakko Abee[a], Sjef Boeren[b], Eddy J. Smid [a], Oscar van Mastrigt[a*]

[a] Food Microbiology, Wageningen University and Research, Wageningen, The Netherlands

[b] Biochemistry, Wageningen University and Research, Wageningen, The Netherlands

*****Corresponding Author: Oscar van Mastrigt, oscar.vanmastrigt@wur.nl

Supplementary Material

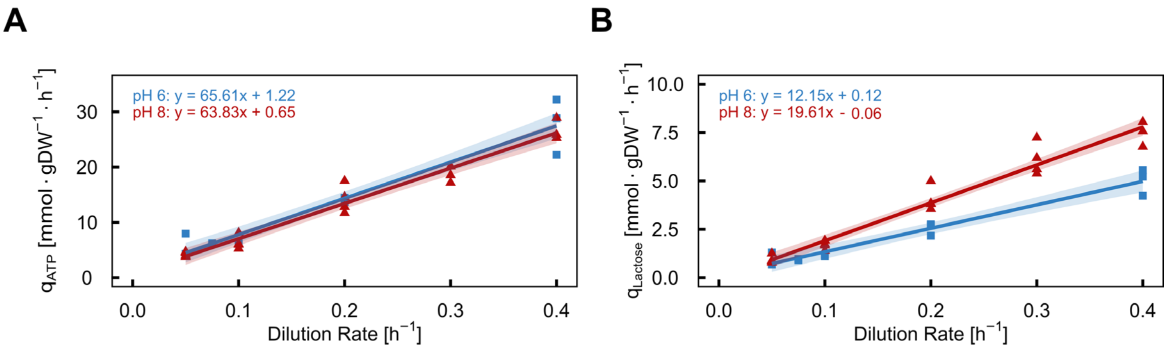

Supplement Figure 1: Relationship between growth rate – set by the dilution rate - and (A) the biomass specific ATP production rate (q_{ATP}) and (B) the specific rate of lactose consumption ($q_{Lactose}$) in a chemostat culture. A linear regression model was fit using the emmeans package in R. The shaded region is the 95 % confidence interval for the regression line. Blue: pH 6 and red: pH 8.

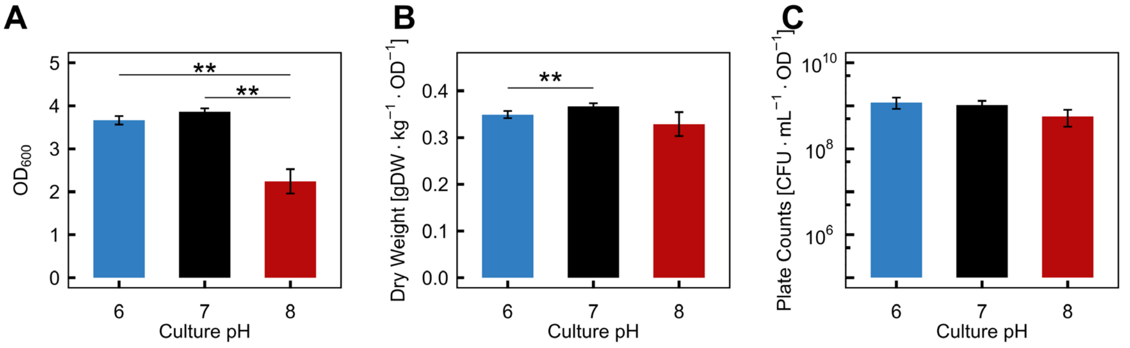

Supplement Figure 2: Effect of pH on culture density in a chemostat culture with a dilution rate of 0.2 h^{-1} grown at pH 6 (blue), pH 7 (black), and pH 8 (red). (A) OD measured at 600 nm. **(B)** Dry weight normalized to OD_{600} in $gDW{\cdot}kg^{-1}{\cdot}OD^{-1}$. **(C)** Culturable cell counts determined by spot plating on LM17 agar plates normalized to OD_{600} in $CFU{\cdot}mL^{-1}{\cdot}OD^{-1}$. The asterisks represents the adjusted statistical significance levels with * = $p \leq 0.05$ and ** = $p \leq 0.01$, calculated using a paired *t*-test with Bonferroni correction. The data was calculated from four independent experiments.

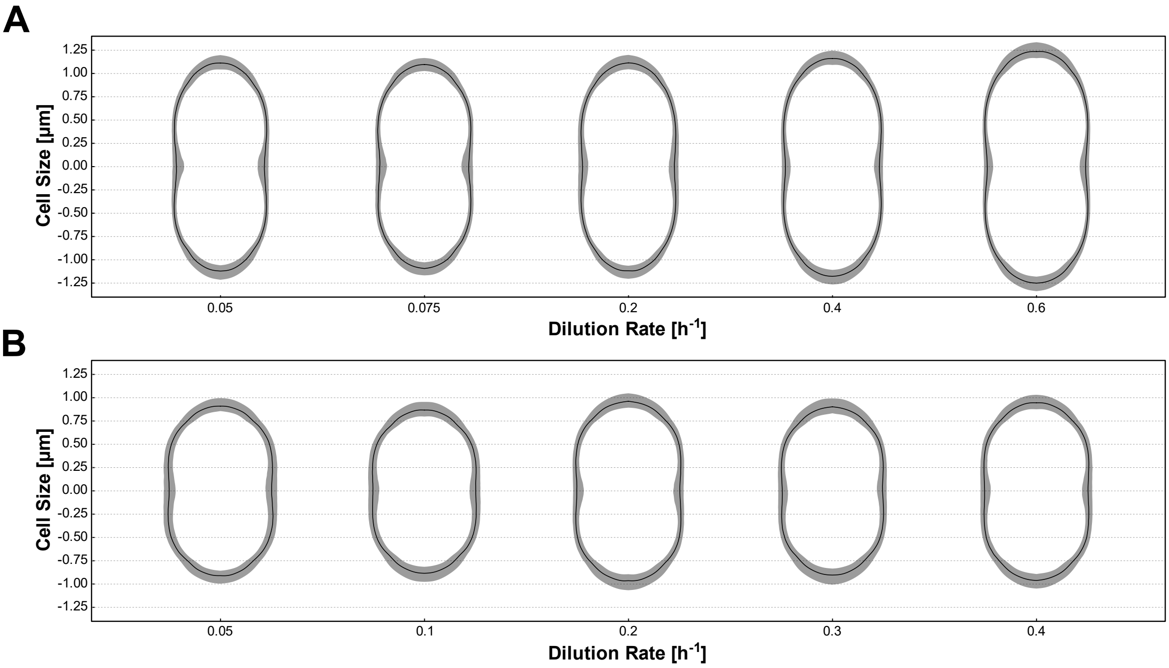

Supplement Figure 3: Average cell shapes in dependence of pH and dilution rate. The plot was created with the Shape Plot function in MicrobeJ based on the data of the category group Chain. The chain length and width is given in µm with the respective standard deviation. Number of cells per dilution rate measured for **(A)** pH 6: 0.05 h^{-1}: n = 906, 1 replicate; 0.075 h^{-1}: n = 1085, 2 replicates; 0.2 h^{-1}: n = 1553, 2 replicates; 0.4 h^{-1}: n =2890, 3 replicates, 0.6 h^{-1}: n = 2009, 3 replicates, **(B)** pH 8: 0.05 h^{-1}: n = 2048, 3 replicates, 0.1 h^{-1}: n = 3069, 3 replicates; n = 0.2 h^{-1}: 2074, 3 replicates; 0.3 h^{-1}: n = 3538, 3 replicates, 0.4 h^{-1}: n = 2287, 3 replicates.

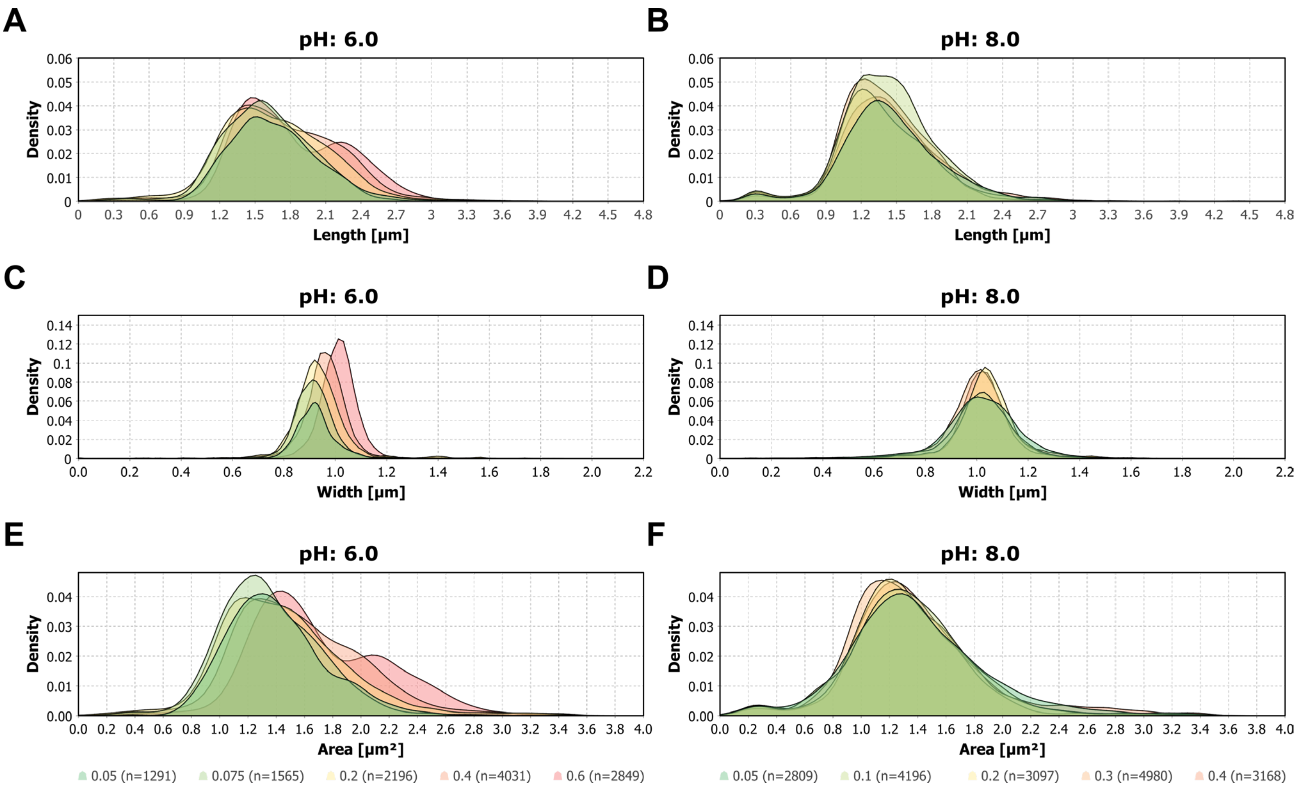

Supplement Figure 4: Density plot showing the distribution of single cell dimensions (length, width, and area) for L. lactis FM03 grown at pH 6 and pH 8 in dependence of dilution rates. The plot was created with the Density Plot function in MicrobeJ based on the data of the category group Cell. **(A)** single cell length of cells grown at pH 6, **(B)**: single cell length of cells grown at pH 8, **(C)** single cell width of cells grown at pH 6, **(D)** single cell width of cells grown at pH 8, **(E)** cell area of cells grown at pH 6, **(F)** cell area of cells grown at pH 8. **pH 6:** dark green: 0.05 h^{-1} (n = 1291, 1 replicate), green: 0.075 h^{-1} (n = 1565, 2 replicates), yellow: 0.2 h^{-1} (n = 2196, 2 replicates), orange: 0.4 h^{-1} (n = 4031, 3 replicates), bright red : 0.6 h^{-1} (n = 2849, 3 replicates), **pH 8:** 0.05 h^{-1}: n = 2809, 3 replicates, 0.1 h^{-1}: n = 4196, 3 replicates; n = 0.2 h^{-1}: 3097, 3 replicates; 0.3 h^{-1}: n = 4980, 3 replicates, 0.4 h^{-1}: n = 3168, 3 replicates.

Supplement Table 1: Occurrence of cell chains with more than two cells in a chemostat culture at a dilution rate of 0.2 h^{-1}. The cell chains were counted manually from at least ten microscopic images per independent chemostat culture and pH.

pH	Biol. replicate	Chain Length (n)						
		**3**	**4**	**5**	**6**	**7**	**8**	**9**
**6**	**1**							
	**2**	2	2	1				
	**3**		1					
**7**	**1**							
	**2**	6	3					
	**3**	10	13	1				
**8**	**1**	10	12	3	5	2	5	
	**2**	5	6	2	1		1	
	**3**	7	14	1	2		2	1

Supplement Table 2: Overview of all quantified proteins in *Lactococcus lactis* across pH 6, 7, and 8 under chemostat conditions (D = 0.2 h^{-1}). For each protein, the table includes functional annotation, log_2-transformed LFQ intensities per condition, ANOVA p-value, adjusted p-values for pairwise comparisons (Tukey p*osthoc* test), and corresponding log_2-fold changes.

Locus Tag	Gene Name	Functional Annotation	ANOVA *p*-value	Adjusted *p*-value			log_2-Fold Change		
				pH 6 - 7	pH 6 - 8	pH 7 - 8	pH 6 - 7	pH 6 - 8	pH 7 - 8
BSR25_RS08050	glcK	ROK family glucokinase	0.456	0.491	0.550	0.99	0.47	-0.04	0.43
BSR25_RS08990	pgi	glucose-6-phosphate isomerase	0.593	0.731	0.969	0.59	0.18	-0.24	-0.06
BSR25_RS04415	pfkA	6-phosphofructokinase	0.139	0.915	0.259	0.15	-0.10	0.50	0.41
BSR25_RS07390	fba	class II fructose-bisphosphate aldolase	0.03	0.17	0.03	0.48	-0.46	-0.74	-0.28
BSR25_RS03335	tpiA	triose-phosphate isomerase	0.83	0.81	0.94	0.96	0.28	0.15	-0.12
BSR25_RS00655	gap1	type I glyceraldehyde-3-phosphate dehydrogenase	0.08	0.99	0.12	0.10	0.02	-0.49	-0.51
BSR25_RS09410	gap2	type I glyceraldehyde-3-phosphate dehydrogenase	0.03	0.18	0.03	0.45	-0.40	-0.65	-0.26
BSR25_RS11375	pgk	phosphoglycerate kinase	0.75	0.89	0.73	0.95	0.09	0.15	0.06
BSR25_RS11850	pgm	phosphoglycerate mutase	0.05	0.85	0.05	0.12	0.19	0.74	0.93
BSR25_RS11530	eno1	phosphopyruvate hydratase	0.00	0.08	0.00	0.00	-1.29	-4.17	-2.88
BSR25_RS01085	eno2	surface-displayed alpha-enolase	0.19	0.53	0.17	0.66	0.43	0.77	0.34
BSR25_RS04410	pyk	pyruvate kinase	0.00	0.01	0.00	0.06	-0.44	-0.76	-0.32
BSR25_RS04405	ldh	L-lactate dehydrogenase	0.40	0.77	0.75	0.37	-0.11	0.11	0.22
BSR25_RS12050	ldhB	L-lactate dehydrogenase	0.08	0.33	0.06	0.52	-0.68	-1.18	-0.51
BSR25_RS09990	pdhA	thiamine pyrophosphate-dependent dehydrogenase E1 component subunit alpha	0.03	0.46	0.03	0.17	-0.53	-1.37	-0.84
BSR25_RS01205	pflB	formate C-acetyltransferase	0.00	0.46	0.00	0.00	-0.33	-2.00	-1.67
BSR25_RS06075	pta	phosphate acetyltransferase	0.05	0.66	0.05	0.18	-0.19	-0.61	-0.42

Table continued on next page

Supplement Table 2 (continued): Overview of all quantified proteins in *Lactococcus lactis* across pH 6, 7, and 8 under chemostat conditions (D = 0.2 h^{-1}). For each protein, the table includes functional annotation, log_2-transformed LFQ intensities per condition, ANOVA p-value, adjusted p-values for pairwise comparisons (Tukey p*osthoc* test), and corresponding log_2-fold changes.

BSR25_RS08925	adhE	acetaldehyde dehydrogenase (acetylating) activity	0.03	0.37	0.02	0.19	0.24	0.56	0.32
BSR25_RS06855	adhP	alcohol dehydrogenase AdhP	0.00	0.93	0.00	0.00	-0.11	-3.76	-3.65
BSR25_RS07995	ackA1	acetate kinase	0.00	0.03	0.00	0.03	-0.66	-1.34	-0.67
BSR25_RS07990	ackA2	acetate kinase	0.00	0.00	0.02	0.08	1.75	1.01	-0.74
BSR25_RS03595	mae	NADP-dependent malic enzyme	0.01	0.01	0.01	1.00	-1.46	-1.43	0.03
BSR25_RS03610	citC	[citrate (pro-3S)-lyase] ligase	0.01	0.10	0.01	0.30	-0.72	-1.22	-0.50
BSR25_RS03615	citD	citrate lyase acyl carrier protein	0.00	0.00	0.00	0.15	-2.46	-3.35	-0.88
BSR25_RS03620	citE	citrate (pro-3S)-lyase subunit beta	0.00	0.00	0.00	0.01	-1.88	-2.94	-1.07
BSR25_RS03625	citF	citrate lyase subunit alpha	0.00	0.00	0.00	0.01	-1.63	-2.60	-0.97
BSR25_RS03630	citG	citrate lyase holo-[acyl-carrier protein] synthase	0.00	0.00	0.00	0.07	-2.02	-2.84	-0.82
BSR25_RS03585	alsS	acetolactate synthase AlsS	0.13	0.14	0.95	0.22	-0.50	-0.07	0.43
BSR25_RS03200	aldC	acetolactate decarboxylase	0.00	0.03	0.00	0.20	-2.15	-3.43	-1.28
BSR25_RS03865	aldB	acetolactate decarboxylase	0.76	0.74	0.95	0.90	-0.14	-0.06	0.08
BSR25_RS02380	butA	(S)-acetoin forming diacetyl reductase	0.00	0.04	0.00	0.00	-0.88	-3.03	-2.15
BSR25_RS02375	butB	2,3-butanediol dehydrogenase	0.00	0.00	0.00	0.00	-2.14	-5.63	-3.48
BSR25_RS06885	pflA	pyruvate formate-lyase-activating protein	0.04	0.35	0.03	0.31	-0.25	-0.52	-0.27
